# Genome sequencing, *de novo* assembly and annotation of the commercially important bamboo, *Bambusa tulda* Roxb

**DOI:** 10.1038/s41597-026-06679-5

**Published:** 2026-02-04

**Authors:** Sutrisha Kundu, Oliver Rupp, Sonali Dey, Mridushree Basak, Sudeshna Bera, Annette Becker, Malay Das

**Affiliations:** 1 Plant Genomics Laboratory, Department of Life Sciences, Presidency University, 86/1 College Street, Kolkata, 700073 West Bengal India; 2 Bioinformatik und Systembiologie, Justus-Liebig-University, Gießen, D-35390 Germany; 3 Institute of Botany, Justus-Liebig-University, Gießen, D-35392 Germany

**Keywords:** Plant sciences, Computational biology and bioinformatics

## Abstract

*Bambusa tulda* Roxb., a member of the Bambusoideae subfamily, is an ecologically and commercially important plant resource widely distributed in the Indian subcontinent. Our study reports long-read PacBio HiFi sequencing and genome assembly of *B. tulda*. The *de novo* haploid genome assembly of *B. tulda* predicted 43 contigs, distributed across three subgenomes, with a total sequenced length of 1.37 Gb, contig N50 of 35.69 Mb, and BUSCO score 99%. Repetitive elements constitute 63.31% of the genome. Functional annotation predicted 56,890 protein-coding genes, constituting 19.44% of the genome. This genome sequence will serve as an invaluable resource for future studies on the life history traits, phylogenomic analysis, comparative genomics, and targeted genome modification for important trait improvement of *B. tulda*.

## Background & Summary

**Fig. 1:**
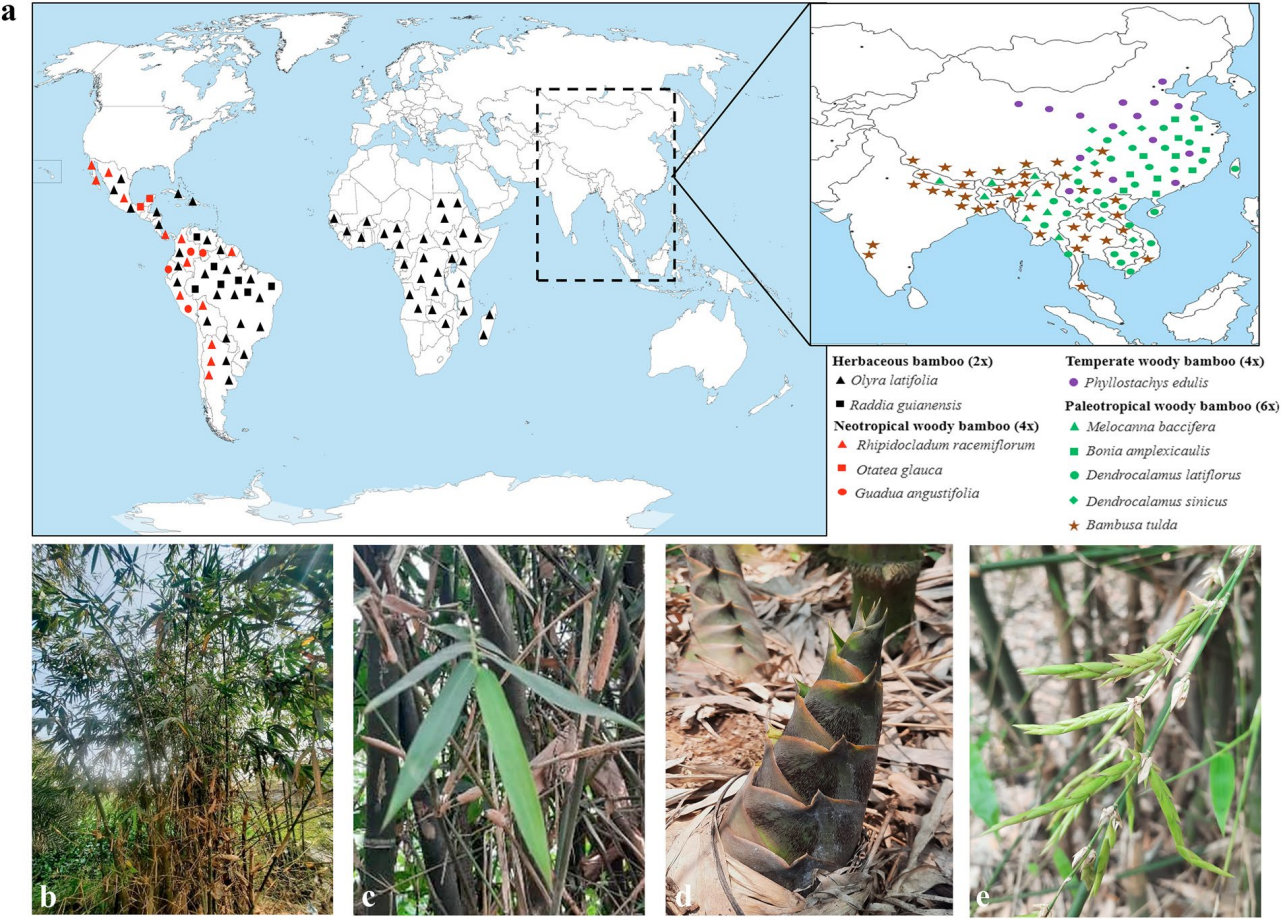
(**a**) Worldwide native distribution of sequenced bamboo genomes, alongwith *B. tulda*. The map was drawn based on information obtained from World Flora Online (WFO 2024), Kew (POWO, 2025), and Guadua Bamboo (https://www.guaduabamboo.com/). (**b**) *Bambusa tulda* plant in native habitat, (**c**) leaf, (**d**) emerging culm, and (**e**) spikelet inflorescence.

Bamboos are among the fastest growing, non-timber, renewable plant resources and are widely distributed throughout the tropics and subtropics^1,2^. They comprise the Bambusoideae subfamily of the monocotyledonous grass family Poaceae, and have significant ecological and economical value^3,4^. The Bambusoideae subfamily is classified into two clades: herbaceous bamboos and woody bamboos^5^. Herbaceous bamboos possess weakly developed rhizomes, lower lignocellulosic biomass, and exhibit annual or seasonal flowering cycles^6^. In contrast, the woody bamboos demonstrate rapid culm growth, high accumulation of lignocellulosic biomass, and delayed flowering subsequent upon lengthy vegetative phases upto 120 years^1,7–9^. Although, the first bamboo genome *Phyllostachys heterocycla* ( = *P*. *edulis*) was sequenced more than a decade ago^10^, major progress in bamboo genome sequencing was achieved more recently due to the introduction of long-read sequencing technologies and advancement in algorithms for genome assembly and annotation^11^. Twelve species from the Bambusoideae subfamily, with diverse worldwide distribution, have been sequenced and their chromosome-level assemblies have been published (Fig. 1a)^2,5^.

*Bambusa tulda*, also known as the Bengal bamboo, is a paleotropical woody bamboo, native to the Indian subcontinent, and certain parts of southeast Asia (Fig. 1). It is putatively aneuploid (70–72 chromosomes)^12^ in nature, and possesses highly lignified culms. Its tentative flowering time is ~50 years^13^ and exhibits both sporadic and gregarious flowering^14^. In addition to their conventional use as construction wood in rural housing in south and southeast Asia, the plant draws renewed attention due to their potential use in the paper-pulp industry^15^ and bio-energy sector^9^. *In vitro* micropropagation method have also been optimized for *B. tulda*, providing opportunity for genetic intervention^16^. Until now, some flowering-associated genes were identified for functional studies in *B. tulda* flowering^17–20^. However, in the absence of a good quality, whole genome sequence, comparative evolutionary studies and genetic manipulation for trait improvement remain challenging.

**Fig. 2:**
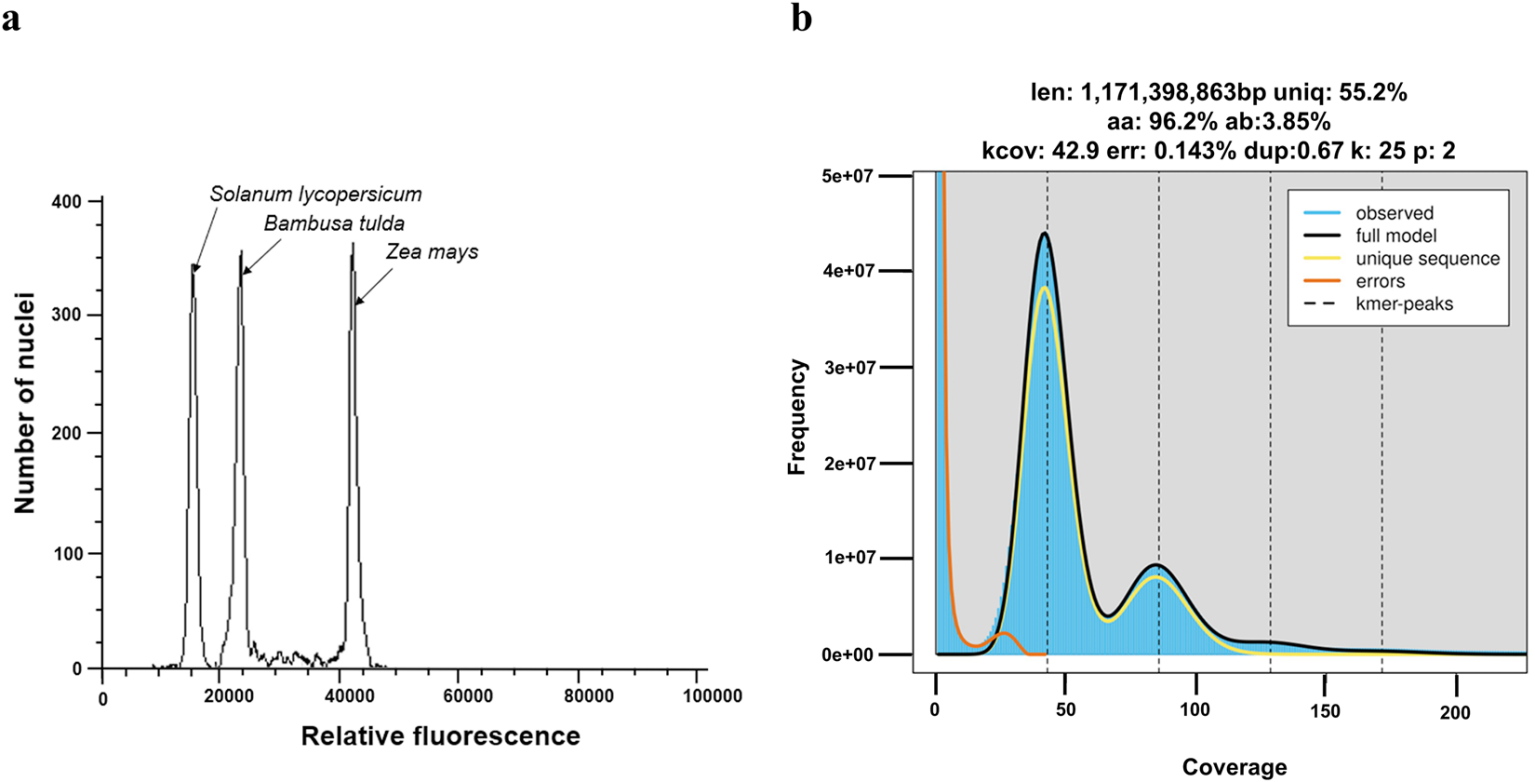
Genome size estimation of *Bambusa tulda*. (**a**) Flow cytometry based estimation of genome size from fluorescence intensity histograms showing mean peak positions of *B. tulda*, *Solanum lycopersicum*, and *Zea mays*. The estimated diploid genome size of *B. tulda* was 3.01 Gb and 3.02 Gb on the basis of the two reference plants *S. lycopersicum* and *Z. mays*, respectively. (**b**) Genome size estimation based on *k*-mer size of 25 using Jellyfish-2 and Genomescope 2.0. The estimated haploid genome size was ~1.17 Gb. Thus, the estimated diploid genome size of *B. tulda* was ~2.34 Gb. The analysis also reveals unique sequences, with both homozygous and heterozygous distribution.

**Fig. 3:**
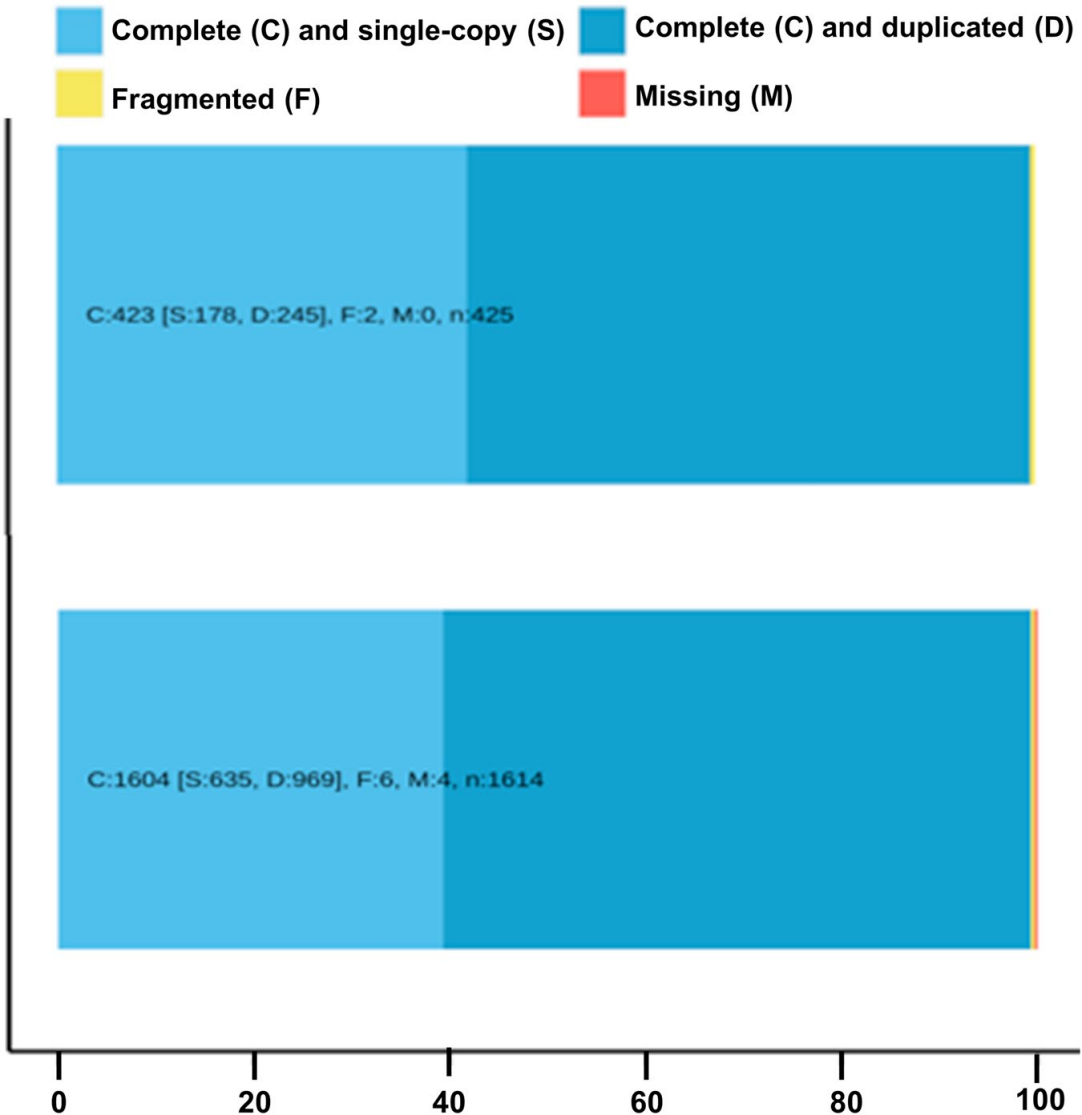
Evaluation of genome assembly completeness of *Bambusa tulda* against the Embryophyte (n = 425) and Viridiplantae (n = 1614) lineage using BUSCO analysis, demonstrating the complete, fragmented, and missing BUSCO groups.

This study aimed to sequence, assemble, and annotate the *B. tulda* genome to provide a genomic resource for this valuable species. Long-read, PacBio high-fidelity (HiFi) sequencing was performed to obtain a genome assembly of *B. tulda*. The diploid genome size estimated by flow cytometry and *k*-mer analyses were ~3 Gb and ~2.34 Gb, respectively (Table 1, Fig. 2). Approximately 116.38  Gb raw PacBio HiFi data and 76.80 Gb transcriptome data were obtained (Tables 2, 3). The primary contig assembly, generated through HiFiasm, produced 752 contigs with the contig N50 value 31.02 Mb (Table 4). Two haplotypes were obtained from a haplotype-resolved assembly, with haplotype 1 containing 827 and haplotype 2 containing 326 contigs (Table 4). Further refinement and mapping of the primary assembly with other sequenced bamboo genomes produced a final haploid genome assembly of 43 contigs with contig N50 value 35.69 Mb (Table 5). Genome assembly quality confirmed a BUSCO score of ~99% (Fig. 3). Genome annotation identified 56,890 protein-coding genes, constituting 19.44% of the genome (Table 6). Repetitive sequences constituted major part (63.31%) of the *B. tulda* genome (Table 7). Additionally, 1,355 rRNA and 1,019 tRNA genes were identified (Table 8).

**Table 1:** Estimation of genome size using flow cytometry analyses.

Sample	Reference	G1 Peak Mean Position of Reference	G1 Peak Mean Position of Sample	Ratio	Diploid Genome Size (Gb)
*Bambusa tulda*	*Solanum lycopersicum*	15,557	24,567	1.58	3.01
*Bambusa tulda*	*Zea mays*	42,980	24,328	0.57	3.02

**Table 2:** Sequencing statistics of *Bambusa tulda* genome.

	DNA1	DNA2	DNA1	DNA2	DNA3	Total
Sequencing Platform	Pacio Revio	PacBio Revio	PacBio Sequel IIe	PacBio Sequel IIe	PacBio Sequel IIe	
Reads	54,80,695	18,73,037	11,02,778	7,34,918	10,72,867	1,02,64,295
Size	54.99 gb	24.64 gb	13.75 gb	9.20 gb	13.80 gb	116.38 gb
Maximum	40.10 kb	43.91 kb	35.37 kb	38.96 kb	38.55 kb	43.91 kb
Mean	10.03 kb	13.16 kb	12.47 kb	12.52 kb	12.86 kb	11.34 kb
Median	10.21 kb	12.72 kb	12.10 kb	12.46 kb	12.37 kb	11.38 kb
Minimum	111.00 bp	89.00 bp	102.00 bp	97.00 bp	138.00 bp	89.00 bp
N50	10.67 kb	13.06 kb	12.51 kb	12.91 kb	12.77 kb	11.87 kb

**Table 3:** Sequencing statistics of *Bambusa tulda* transcriptome.

Sequencing Platform	Illumina NovaSeq 600 PE150
**Raw Reads**	51,17,96,852
**Raw Data**	76.80 Gb
**Effective Reads**	99.52%
**Base Error Rate**	0.03%
**Q20**	97.41%
**Q30**	93.08%
**GC Content**	53.02%

**Table 4:** Assembly statistics of the primary and haplotype-resolved *Bambusa tulda* genome.

	Primary Assembly	Haplotype 1	Haplotype 2
**Contigs**	752	827	326
**Size**	1.85 Mb	1.38 Gb	1.40 Gb
**Maximum**	63.43 Mb	57.40 Mb	63.43 Mb
**Mean**	2.46 Mb	1.67 Mb	4.30 Mb
**Median**	30.58 kb	30.15 kb	49.41 kb
**Minimum**	4.40 kb	4.40 kb	15.70 kb
**N50**	31.02 Mb	26.77 Mb	28.84 Mb
**L50**	22	19	18
**N90**	15.11 Mb	8.87 Mb	10.98 Mb
**L90**	52	50	47

**Table 5:** Assembly statistics of the final genome of *Bambusa tulda*.

	Final assembly
Contigs	43
Size	1.37 Gb
Maximum	63.43 Mb
Mean	31.77 Mb
Median	30.48 Mb
Minimum	10.51 Mb
N50	35.69 Mb
L50	15
GC Content	44.61%

**Table 6:** Statistics of gene prediction in *Bambusa tulda* genome.

Property	Value
Total Gene Length	26,55,70,276 bp
Total Gene Number	56,890
Genes Percentage of Genome	19.44%
Total Exon Length	17,70,27,591 bp
Average Exons per Gene	5.88
Exon Percentage of Genome	12.96%
Total CDS Length	11,64,29,828 bp
Total CDS Number	89,479
CDS Percentage of Genome	8.52%
Average Gene Length	4,668.14 bp
Average Exon Length	336.63 bp
Average CDS Length	240.03 bp

**Table 7:** Statistics of repeat elements in *Bambusa tulda* genome.

Type of Repeat Element			Number	Length occupied (bp)	Percentage of sequence used
Retroelements			4,70,206	37,08,35,520	27.15%
	SINEs		2,229	6,56,608	0.05%
	Penelope		544	1,42,470	0.01%
	LINEs		19,403	1,14,94,431	0.84%
		CRE/SLACS	0	0	0.00%
		L2/CR1/Rex	0	0	0.00%
		R1/LOA/Jockey	0	0	0.00%
		R2/R4/NeSL	0	0	0.00%
		RTE/Bov-B	3,393	18,96,413	0.14%
		L1/CIN4	16,010	95,98,018	0.70%
	LTR Elements		4,48,574	35,86,84,481	26.26%
		BEL/Pao	0	0	0.00%
		Ty1/Copia	1,57,741	15,30,30,961	11.20%
		Gypsy/DIRS1	1,78,327	16,66,69,779	12.20%
		Retroviral	1,591	3,54,427	0.03%
DNA Transposons			1,35,412	8,44,57,392	6.18%
		Hobo-Activator	29,216	1,30,12,505	0.95%
		Tc1-IS630-Pogo	0	0	0.00%
		En-Spm	0	0	0.00%
		MULE-MuDR	3,55,055	2,29,17,028	1.68%
		PiggyBac	0	0	0.00%
		Tourist/Harbinger	7,219	30,90,101	0.23%
		Other (Mirage, P-element, Transib)	0	0	0.00%
Rolling-circles			6,674	47,62,233	0.35%
Unclassified			14,18,434	40,94,03,385	29.97%
Total Interspersed Repeats				86,48,38,767	63.31%
Small RNA			2,052	5,83,211	0.04%
Satellites			790	2,11,000	0.02%
Simple Repeats			1,65,904	86,65,086	0.63%
Low Complexity			22,659	13,53,496	0.10%

**Table 8:** Statistics of non-coding rRNA and tRNA prediction in *Bambusa tulda* genome.

Type of ncRNA	Count	Total Length (bp)	Average Length (bp)	Percentage of Genome
18S rRNA	15	19,703	1,313.53	0.001442%
28S rRNA	17	31,624	1,860.24	0.002315%
5.8S rRNA	8	1,223	152.88	0.000090%
5S rRNA	1,315	1,51,008	114.83	0.011054%
tRNA	1,019	77,003	75.57	0.005637%

*B. tulda* shows interesting life history traits but the genetic regulation of flowering time delay and how rapid vegetative growth is promoted remains unclear. Moreover, with this genome sequence, comparative genomics can uncover the polyploidization history of *B. tulda*, resolve major gene family expansions/contractions and repeat element evolution in the bamboo lineages.

## Materials and Methods

### Sample collection

Tissues were collected from a natural, flowering population of *B. tulda*, located at Rahuta, Shyamnagar (22.83°N, 88.40°E), West Bengal, India. Fresh young leaves collected were used for genome size estimation and whole genome sequencing, while for transcriptome sequencing, emerging culm was used (Fig. 1b–d). All the collected tissues were quickly flash-frozen in dry ice, and subsequently stored at −80 °C.

### Genome size estimation by flow cytometry analysis

For genome size estimation, two plants with known genome sizes: *Solanum lycopersicum* L. Stupicke´ polnı´ rane´ (1.96 pg/2 C DNA) and *Zea mays* L. ‘CE-777’ (5.43 pg/2 C DNA) were used as internal references. Isolation of nuclei was carried out by following the nuclei-isolation protocol of Dolezel^21^. In brief, 40 mg fresh young leaf tissue from both the reference plants and *B. tulda* were finely chopped in 1 ml ice-cold nuclei isolation Galbraith buffer. The homogenate was filtered through a 40 μm nylon mesh. The filtrate was treated simultaneously with 50 μg/ml RNase A and 50 μg/ml propidium iodide. After incubation in the dark for 30 mins, the suspension of stained nuclei was introduced into flow cytometer (S3e Cell Sorter, Bio-Rad), using argon-ion laser of 488 nm. Live gating was set around the fluorescence area signal (FL3-A) to obtain cell cycle histograms. Based on the fluorescence histograms, the nuclear DNA content was calculated. The estimated diploid genome size of *B. tulda* was 3.01 Gb and 3.02 Gb on the basis of the two reference plants *S. lycopersicum* and *Z. mays*, respectively (Fig. 2a, Table 1).

### Genome and transcriptome sequencing

High molecular weight (HMW) genomic DNA was extracted from the young leaves using the NucleoBond® HMW DNA kit (Macherey-Nagel, Germany), following the manufacturer’s protocol. The extracted HMW genomic DNA was fragmented to obtain Single-Molecule Real-Time (SMRT) bell libraries. The SMRTbell libraries were then sequenced in the PacBio Sequel IIe (3 libraries) and PacBio Revio (2 libraries) platform (Novogene, Genomics Singapore Pte. Ltd) (Fig. S1, S2). The raw reads obtained by sequencing 5 libraries were pooled together and subjected to quality control. Genome sequencing in the PacBio Sequel IIe platform yielded 36.75 Gb data with N50 value of 12.73 kb and mean read length 12.62 kb (Table 2). Similarly, genome sequencing in the PacBio Revio platform yielded 79.63 Gb data with N50 value of 11.87 kb and mean read length 11.60 kb and (Table 2). From the two sequencing platforms, a total of 1,02,64,295 PacBio SMRT reads corresponding to 116.38 Gb data was obtained (Table 2).

For accurate annotation of the *B. tulda* genome, transcriptome sequencing was performed in the Illumina Novaseq. 6000 platform. Total RNA was extracted from tissues using the RNeasy® Plant Mini Kit (Qiagen), following the manufacturer’s protocol. The integrity of isolated RNAs was examined in the Agilent 5400 Bioanalyzer, and sample with RNA integrity value > 8 were used for library construction. The library was subjected to the Illumina NovaSeq. 6000 PE150 platform for sequencing. Transcriptome sequencing yielded a total raw data output of 76.80 Gb with a Q30 value of 93.08% (Table 3).

### Estimation of genome characteristics

Following genome sequencing, *k*-mer analysis was performed to estimate the genome size, heterozygosity rate, and proportion of repetitive sequences of *B. tulda*. The distribution of *k*-mer frequency from the raw PacBio HiFi reads was performed in Jellyfish (v2.3.0)^22^ software. The *k*-mer spectrum obtained for “-*k* 25”, ploidy = 2 was then analyzed using the GenomeScope 2.0^23^ web tool. The predicted haploid genome size was ~1.17 Gb, with a substantial proportion of repetitive sequences (44.80%), heterozygosity rate 3.85%, and sequencing error rate 0.14% (Fig. 2b). Smaller peaks at 3x and 4x coverage indicated possible genome duplication. Thus, the estimated diploid genome size of *B. tulda* from this analysis was ~2.34 Gb. The estimated genome size using k = 25 was smaller than that obtained by flow cytometry due to the challenge of assembling the high portion of repetitive fraction of the genome, and can also be affected by heterozygosity, polyploidy, and possibly genome duplication.

### *De novo* genome assembly

The clean PacBio HiFi reads were assembled *de novo* into a haplotype-resolved assembly and a primary contig assembly (a combination of both haplotypes) using Hifiasm v0.16.1-R341^24^ tool. The primary contig assembly produced a total of 752 contigs equivalent to 1.85 Gb of data with the N50 value 31.02 Mb (Table 4, Fig. S3a). Size of the two haplotype assemblies were 1.38 Gb for Haplotype 1 and 1.40 Gb for Haplotype 2 (Table 4, Fig. S3b).

**Fig. 4:**
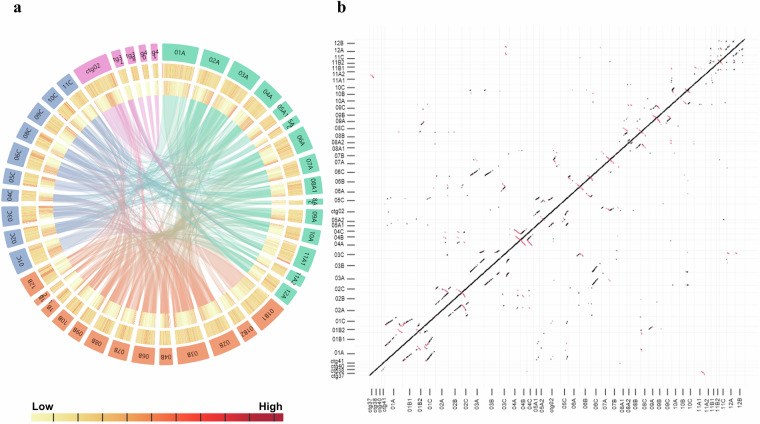
Genomic features for the 43 contigs of the *Bambusa tulda* genome. (**a**) Circos plot representing the different genome features of the *B. tulda* genome assembly. The outer to inner tracks represent different contigs, GC content, transposable element density, and collinear blocks. Green, orange, and blue blocks represent contigs of subgenome A, subgenome B, and subgenome C, respectively. Purple blocks represent unidentified contigs. The plot was visualized using Accusyn software^53^. (**b**) Dot plot representing the subgenome syntenic relationship of *B. tulda* based on the annotated coding genes.

The 752 contigs from the raw primary assembly were filtered to remove duplicated contigs and contigs from chloroplast and mitochondrial genome. Finally, 43 contigs were obtained which represent the assembled haploid genome sequence of *B. tulda* (Fig. 4a). Among them, 38 contigs were named and divided into three distinct (A, B, and C) subgenomes based on their mapping and homology to genome assemblies of other bamboo species. The remaining five contigs were not named yet. The final haploid genome assembly containing 43 contigs was 1.37 Gb in size, with contig N50 value being 35.69 Mb, mean contig length 31.77 Mb, and GC content was 44.61% (Table 5).

### Annotation of repetitive elements

To annotate repetitive elements in *B. tulda* genome, a combination of *de novo* and homology-based prediction was used. For *de novo* prediction, RepeatModeler^25,26^ was used, which in turn uses two *ab initio* repeat sequence prediction softwares: RECON (v1.0.8)^27^ and RepeatScout (v1.0.6)^28^. LTR_FINDER^29^ was used specifically for *de novo* prediction of LTR elements. Homology-based method to identify the transposable elements involved using the RepeatMasker^30,31^ software against Repbase database.

Approximately, 63.31% of the total *B. tulda* genome was annotated as repeat elements. The identified transposable elements were further classified into two broad categories: Class I retrotransposons and Class II DNA transposons. They represented 27.15% (370 MB) and 6.18% (84 MB) of repetitive sequences, respectively, while 29.97% repeat elements remained unclassified (Table 7, Fig. 4a).

### Gene prediction and functional annotation

For predicting protein-coding genes, a combination of three different methods were applied together: *de novo*, homology-based, and transcriptome-assisted. The *de novo* prediction was done using RNA-Seq and protein evidence data, allowing BRAKER3^32^ to perform automated genome annotation. GeMOMa v1.7^33^ was used for homology-based prediction, by mapping the previously sequenced bamboo gene models^5^. The transcriptome guided genome annotation was conducted using Trinity (v2.1.1)^34,35^, HISAT2 (v2.2.1)^36^, and StringTie (v1.3.3)^37,38^. The transcriptome data was mapped to the genome using HISAT2 to generate BAM files, which were then used as input for genome-guided transcriptome assembly in StringTie. Also, unigene assemblies were generated by *de novo* assembly of transcriptome data using Trinity and rnaSPADES^39^. The genes predicted from all three above methods were consolidated together using Evidence Modeler (EVM) v2.1.0^40^ and PASA^41,42^ to finally obtain non-redundant protein-coding gene structures. In total, 56,890 protein-coding genes constituting 19.44% of the *B. tulda* genome were identified (Table 6). The predicted genes of *B. tulda* were mapped back to the *B. tulda* genome using GeMoMa, with the “synteny checker” module enabled. The dot plot was created with the “synplot.r” script (k = 3) included in GeMoMa on the reference gene table created by the GeMoMa run (Fig. 4b).

For functional annotation, homology-based annotation was done using SwissProt and TrEMBL databases. The predicted protein-coding sequences were aligned against these two databases through BLASTP^43^ analysis with e-value ≤ 1e^−5^. The functional domains of the proteins were identified using InterProScan^44^.

### Annotation of rRNA and tRNA

**Fig. 5:**
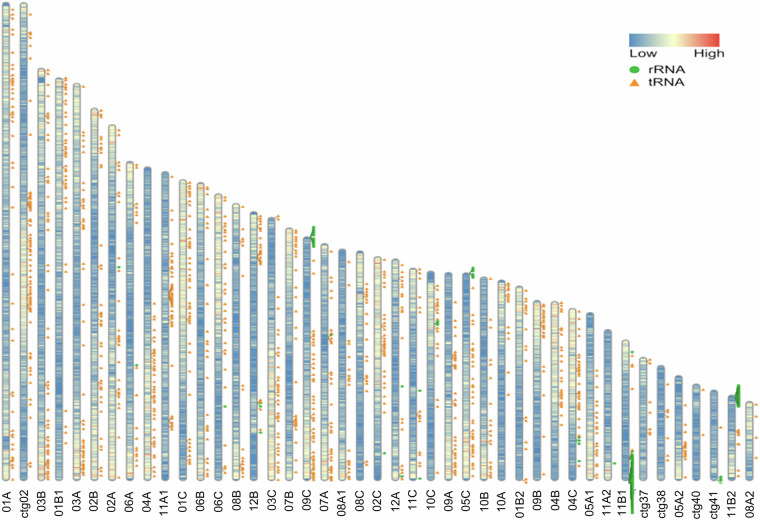
RIdeogram plot to visualize genome-wide gene density, tRNA position, and rRNA position across the 43 assembled contigs of *Bambusa tulda*. The RIdeogram software was used for visualizing the genome features^54^.

Among the non-coding RNA genes, rRNAs and tRNAs were predicted using the *B. tulda* genome. The rRNA genes were predicted using Barrnap v0.9^45^, while the tRNA genes were predicted using tRNAscan-SE^46,47^. From this prediction, 1,355 rRNA and 1,019 tRNA genes were identified successfully (Table 8, Fig. 5).

## Data Records

The raw PacBio genome sequencing data and raw Illumina RNA-Seq data were deposited in the EMBL-EBI European Nucleotide Archive (ENA) database under BioProject accession number ERP174591^48^. The genome assembly, containing 43 contigs and annotation were submitted in GenBank under the accession number GCA_965643865^49^. The genome annotation file in GFF format alongwith the CDS and protein sequences are also available in the Figshare database (10.6084/m9.figshare.31037047)^50^.

## Data Overview

### Phylogenetic analysis

**Fig. 6:**
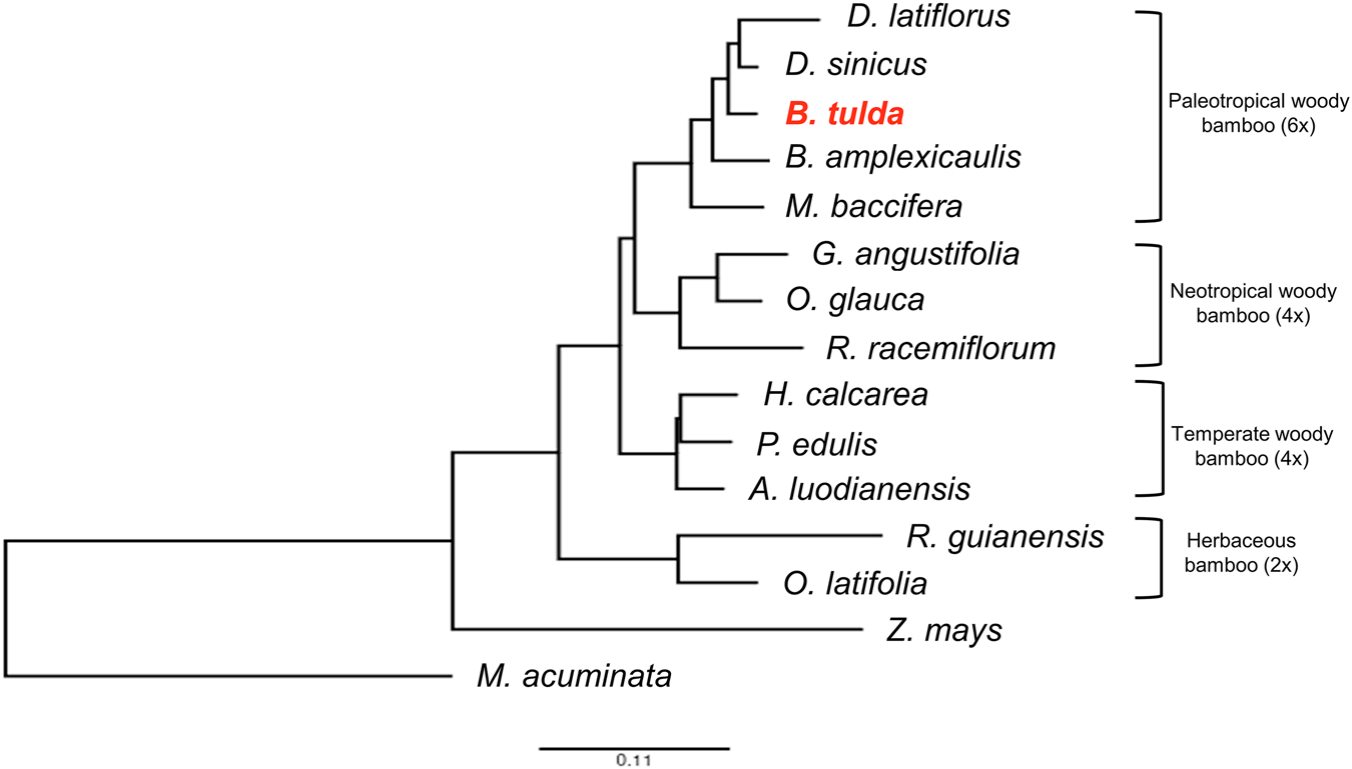
Species tree of *Bambusa tulda* and other sequenced bamboos, with *Zea mays* and *Musa acuminata* used as outgroups.

To determine the evolutionary relationship of *B. tulda* with other monocots, the genomes of twelve bamboo species (*Ampelocalamus luodianensis*, *Bonia amplexicaulis*, *Dendrocalamus latiflorus*, *D. sinicus*, *Guadua angustifolia*, *Hsuehochloa calcarean*, *Melocanna baccifera*, *Olyra latifolia*, *Otatea* glauca, *P. edulis*, *Raddia guianensis*, and *Rhipidocladum racemiflorum*) along with *Z. mays* and *Musa acuminata* (banana), were used to construct a species tree. OrthoFinder^51^ was used to calculate phylogeny reconstructions using the protein sequences of *B. tulda*, the twelve sequenced bamboos, and the two outgroups, *Z. mays* and *M. acuminata* as input data. 202 orthogroups were identified using OrthoFinder, which were used to construct the species tree (Fig. 6). The phylogeny reconstruction confirms that *B. tulda* is member of the hexaploidy paleotropic woody bamboos, which form a monophyletic group.

## Technical Validation

### Quality assessment of high molecular weight genomic DNA, libraries and sequence data

HMW genomic DNA was extracted to subject it for sequencing in the PacBio platform. The quantity and quality of the extracted DNA was examined by qubit fluorometer, and agarose gel (1%) electrophoresis (Fig. S1a). After ensuring required quality, three SMRTbell libraries were constructed from the genomic DNA, with relative fluorescence units (RFU) peaks obtained at 12,567 bp (DNA1), 11,447 bp (DNA2), and 11,307 bp (DNA3) (Fig. S1b–d). Each of these 3 libraries were sequenced in the PacBio Sequel IIe platform, while libraries DNA1 and DNA2 were also sequenced in the PacBio Revio platform. The mean read lengths obtained in the Sequel IIe platform were 12,472 bp (DNA1), 12,524 bp (DNA2), and 12,864 bp (DNA3), while in the Revio platform were 10,033 bp (DNA1) and 13,155 bp (DNA2) (Fig. S2).

### Assessment of genome assembly and annotation completeness

The completeness of the assembled genome was examined in BUSCO v5.8.0^52^ against the Embryophyte (n = 425) and Viridiplantae (n = 1,614) datasets. BUSCO analysis demonstrated 99.0% complete BUSCO groups against both Embryophyte, and Viridiplantae (Fig. 3).

## Supplementary information

Supplementary Figures

## Data Availability

The complete dataset for *B. tulda* whole genome sequencing and assembly are available in the EMBL-EBI ENA database. The raw PacBio genome sequencing data and raw Illumina RNA-Seq data are available under BioProject accession number ERP174591, while the genome assembly and annotation are available at GenBank under accession GCA_965643865. The genome annotation files of *Bambusa tulda* are also available in the Figshare database (10.6084/m9.figshare.31037047).

## Code availability

All softwares and pipeline utilized in this study for data analyses were implemented in full compliance with the manuals and protocols described by the respective published bioinformatics tools. Details on the software versions and parameters are outlined in the Methods section. In cases where specific parameters are not specified, default settings were applied. No custom programming or coding was employed.

## References

[1] Basak, M. *et al*. Genomic insights into growth and development of bamboos: what have we learnt and what more to discover? *Trees* **35**, 1771–1791, 10.1007/s00468-021-02197-6 (2021).

[2] Zheng, Y. *et al*. Allele‐aware chromosome‐scale assembly of the allopolyploid genome of hexaploid Ma Bamboo (*Dendrocalamus latiflorus* Munro). *J. Integr. Plant Biol.* **64**(3), 649–670, 10.1111/jipb.13217 (2022). 34990066 10.1111/jipb.13217

[3] Kellogg, E. A. & Kellogg, E. A. Description of the Family, Vegetative Morphology and Anatomy: Poaceae (R. Br.) Barnh (1895). Gramineae Juss.(1789). *Flowering Plants. Monocots: Poaceae. Cham: Springer International Publishing*, 3–23, 10.1007/978-3-319-15332-2_1 (2015).

[4] Zhao, H. *et al* Bamboo and rattan: Nature-based solutions for sustainable development. *The Innovation*, **3**(6), 10.1016/j.xinn.2022.100337 (2022). 10.1016/j.xinn.2022.100337 PMC9637970 36353673

[5] Ma, P. F. *et al*. Genome assemblies of 11 bamboo species highlight diversification induced by dynamic subgenome dominance. *Nat. Genet.* **56**(4), 710–720, 10.1038/s41588-024-01683-0 (2024). 38491323 10.1038/s41588-024-01683-0 PMC11018529

[6] Li, W. *et al*. Draft genome of the herbaceous bamboo *Raddia distichophylla*. *G3: Genes|Gnomes|Genetics* **11**(2), jkaa049, 10.1093/g3journal/jkaa049 (2021). 33585868 10.1093/g3journal/jkaa049 PMC8022951

[7] Janzen, D. H. Why bamboos wait so long to flower. *Ann. Rev. Ecol. Sys*. 347–391, 10.1146/annurev.es.07.110176.002023 (1976).

[8] Chakraborty, S. *et al*. Studies on reproductive development and breeding habit of the commercially important bamboo *Bambusa tulda* Roxb. *Plants* **10**(11), 2375, 10.3390/plants10112375 (2021). 34834738 10.3390/plants10112375 PMC8619091

[9] Biswas, S. *et al*. Cellulose and lignin profiling in seven, economically important bamboo species of India by anatomical, biochemical, FTIR spectroscopy and thermogravimetric analysis. *Biomass and Bioenergy* **158**, 106362, 10.1016/j.biombioe.2022.106362 (2022).

[10] Peng, Z. *et al*. The draft genome of the fast-growing non-timber forest species moso bamboo (*Phyllostachys heterocycla*). *Nat. Genet.* **45**(4), 456–461, 10.1038/ng.2569 (2013). 23435089 10.1038/ng.2569

[11] Espinosa, E., Bautista, R., Larrosa, R. & Plata, O. Advancements in long-read genome sequencing technologies and algorithms. *Genomics*, 110842, 10.1016/j.ygeno.2024.110842 (2024). 10.1016/j.ygeno.2024.110842 38608738

[12] Kumar, P. P., Turner, I. M., Nagaraja Rao, A. & Arumuganathan, K. Estimation of nuclear DNA content of various bamboo and rattan species. *Plant Biotechnol. Rep.* **5**, 317–322, 10.1007/s11816-011-0185-0 (2011).

[13] Ram, H. M. & Gopal, B. H. Some observations on the flowering of bamboos in Mizoram. *Curr. Sci*., 708–710 (1981).

[14] Bhattacharya, S., Das, M., Bar, R. & Pal, A. Morphological and molecular characterization of *Bambusa tulda* with a note on flowering. *Ann. Bot.* **98**(3), 529–535, 10.1093/aob/mcl143 (2006). 16845134 10.1093/aob/mcl143 PMC2803566

[15] Das, M. & Bhattacharya, S. & Pal, A. Generation and characterization of SCARs by cloning and sequencing of RAPD products: a strategy for species-specific marker development in bamboo. *Ann. Bot.* **95**(5), 835–841, 10.1093/aob/mci088 (2005). 15731116 10.1093/aob/mci088 PMC4246737

[16] Das, M. & Pal, A. Clonal propagation and production of genetically uniform regenerants from axillary meristems of adult bamboo. *J. Plant Biochem. Biotechnol.* **14**, 185–188, 10.1007/BF03355956 (2005).

[17] Biswas, P., Chakraborty, S., Dutta, S., Pal, A. & Das, M. Bamboo flowering from the perspective of comparative genomics and transcriptomics. *Front. Plant Sci.* **7**, 1900, 10.3389/fpls.2016.01900 (2016). 28018419 10.3389/fpls.2016.01900 PMC5156695

[18] Dutta, S. *et al*. Identification, characterization and gene expression analyses of important flowering genes related to photoperiodic pathway in bamboo. *BMC Genomics* **19**, 1–19, 10.1186/s12864-018-4571-7 (2018). 29523071 10.1186/s12864-018-4571-7 PMC5845326

[19] Dutta, S. *et al*. Identification and functional characterization of two bamboo FD gene homologs having contrasting effects on shoot growth and flowering. *Sci. Rep.* **11**(1), 7849, 10.1038/s41598-021-87491-6 (2021). 33846519 10.1038/s41598-021-87491-6 PMC8041875

[20] Basak, M., Chakraborty, S., Kundu, S., Dey, S. & Das, M. Identification, expression analyses of APETALA1 gene homologs in *Bambusa tulda* and heterologous validation of BtMADS14 in Arabidopsis thaliana. *Physiol. Mol. Biol. Plants*, 1–16, 10.1007/s12298-025-01569-3 (2025). 10.1007/s12298-025-01569-3 PMC12006657 40256271

[21] Doležel, J., Greilhuber, J. & Suda, J. Estimation of nuclear DNA content in plants using flow cytometry. *Nat. Protoc.* **2**(9), 2233–2244, 10.1038/nprot.2007.310 (2007). 17853881 10.1038/nprot.2007.310

[22] Marçais, G. & Kingsford, C. A fast, lock-free approach for efficient parallel counting of occurrences of k-mers. *Bioinformatics* **27**(6), 764–770, 10.1093/bioinformatics/btr011 (2011). 21217122 10.1093/bioinformatics/btr011 PMC3051319

[23] Ranallo-Benavidez, T. R., Jaron, K. S. & Schatz, M. C. GenomeScope 2.0 and Smudgeplot for reference-free profiling of polyploid genomes. *Nat. Commun.* **11**(1), 1432, 10.1038/s41467-020-14998-3 (2020). 32188846 10.1038/s41467-020-14998-3 PMC7080791

[24] Cheng, H., Concepcion, G. T., Feng, X., Zhang, H. & Li, H. Haplotype-resolved de novo assembly using phased assembly graphs with hifiasm. *Nat. Methods* **18**(2), 170–175, 10.1038/s41467-020-14998-3 (2021). 33526886 10.1038/s41592-020-01056-5 PMC7961889

[25] Smit, A. & Hubley, R. RepeatModeler Open-1.0. Available online at: https://www.repeatmasker.org (2015).

[26] Flynn, J. M. *et al*. RepeatModeler2 for automated genomic discovery of transposable element families. *Proc. Natl. Acad. Sci. USA* **117**(17), 9451–9457, 10.1073/pnas.1921046117 (2020). 32300014 10.1073/pnas.1921046117 PMC7196820

[27] Bao, Z. & Eddy, S. R. Automated de novo identification of repeat sequence families in sequenced genomes. *Genome Res.* **12**(8), 1269–1276, http://www.genome.org/cgi/doi/10.1101/gr.88502 (2002). 12176934 10.1101/gr.88502 PMC186642

[28] Price, A. L., Jones, N. C. & Pevzner, P. A. De novo identification of repeat families in large genomes. *Bioinformatics* **21**(suppl_1), i351–i358, 10.1093/bioinformatics/bti1018 (2005). 15961478 10.1093/bioinformatics/bti1018

[29] Xu, Z. & Wang, H. LTR_FINDER: an efficient tool for the prediction of full-length LTR retrotransposons. *Nucleic Acids Res.* **35**(suppl_2), W265–W268, 10.1093/nar/gkm286 (2007). 17485477 10.1093/nar/gkm286 PMC1933203

[30] Tarailo-Graovac, M. & Chen, N. Using RepeatMasker to identify repetitive elements in genomic sequences. *Curr. Protoc. Bioinformatics*, Chapter 4, 4.10. 1–4.10. 14, 10.1002/0471250953.bi0410s05 (2009). 10.1002/0471250953.bi0410s25 19274634

[31] Smit, A. F. A., Hubley, R. & Green, P. RepeatMasker Open-4.0. Available online at: https://www.repeatmasker.org (2013-2015).

[32] Gabriel, L. *et al*. BRAKER3: Fully automated genome annotation using RNA-seq and protein evidence with GeneMark-ETP, AUGUSTUS, and TSEBRA. *Genome Res.* **34**(5), 769–777, https://www.genome.org/cgi/doi/10.1101/gr.278090.123 (2024). 38866550 10.1101/gr.278090.123 PMC11216308

[33] Keilwagen, J., Hartung, F. & Grau, J. GeMoMa: homology-based gene prediction utilizing intron position conservation and RNA-seq data. *Gene prediction**:**Methods in Mol. Biol*. **1962**, Humana, New York, NY, 161–177, 10.1007/978-1-4939-9173-0_9 (2019). 10.1007/978-1-4939-9173-0_9 31020559

[34] Grabherr, M. G. *et al*. Trinity: reconstructing a full-length transcriptome without a genome from RNA-Seq data. *Nat. Biotechnol.* **29**(7), 644, 10.1038/nbt.1883 (2011). 21572440 10.1038/nbt.1883 PMC3571712

[35] Haas, B. J. *et al*. De novo transcript sequence reconstruction from RNA-seq using the Trinity platform for reference generation and analysis. *Nat. Protoc.* **8**(8), 1494–1512, 10.1038/nprot.2013.084 (2013). 23845962 10.1038/nprot.2013.084 PMC3875132

[36] Kim, D., Langmead, B. & Salzberg, S. L. HISAT: a fast spliced aligner with low memory requirements. *Nat. Methods* **12**(4), 357–360, 10.1038/nmeth.3317 (2015). 25751142 10.1038/nmeth.3317 PMC4655817

[37] Pertea, M. *et al*. StringTie enables improved reconstruction of a transcriptome from RNA-seq reads. *Nat. Biotechnol.* **33**(3), 290–295, 10.1038/nbt.3122 (2015). 25690850 10.1038/nbt.3122 PMC4643835

[38] Kovaka, S. *et al*. Transcriptome assembly from long-read RNA-seq alignments with StringTie2. *Genome Biol.* **20**, 1–13, 10.1186/s13059-019-1910-1 (2019). 31842956 10.1186/s13059-019-1910-1 PMC6912988

[39] Bushmanova, E., Antipov, D., Lapidus, A. & Prjibelski, A. D. rnaSPAdes: a de novo transcriptome assembler and its application to RNA-Seq data. *GigaScience* **8**(9), giz100, 10.1093/gigascience/giz100 (2019). 31494669 10.1093/gigascience/giz100 PMC6736328

[40] Haas, B. J. *et al*. Automated eukaryotic gene structure annotation using EVidenceModeler and the Program to Assemble Spliced Alignments. *Genome Biol.* **9**, 1–22, 10.1186/gb-2008-9-1-r7 (2008). 10.1186/gb-2008-9-1-r7 PMC2395244 18190707

[41] Haas, B. J. *et al*. Improving the Arabidopsis genome annotation using maximal transcript alignment assemblies. *Nucleic Acids Res.* **31**(19), 5654–5666, 10.1093/nar/gkg770 (2003). 14500829 10.1093/nar/gkg770 PMC206470

[42] Campbell, M. A., Haas, B. J., Hamilton, J. P., Mount, S. M. & Buell, C. R. Comprehensive analysis of alternative splicing in rice and comparative analyses with Arabidopsis. *BMC Genomics* **7**, 1–17, 10.1186/1471-2164-7-327 (2006). 17194304 10.1186/1471-2164-7-327 PMC1769492

[43] Altschul, S. F. *et al*. & Lipman, D. J. Gapped BLAST and PSI-BLAST: a new generation of protein database search programs. *Nucleic Acids Res.* **25**(17), 3389–3402, 10.1093/nar/25.17.3389 (1997). 9254694 10.1093/nar/25.17.3389 PMC146917

[44] Jones, P. *et al*. InterProScan 5: genome-scale protein function classification. *Bioinformatics* **30**(9), 1236–1240, 10.1093/bioinformatics/btu031 (2014). 24451626 10.1093/bioinformatics/btu031 PMC3998142

[45] Seemann, T. barrnap 0.9: rapid ribosomal RNA prediction. *Google Scholar*, *792*. Available online at https://github.com/tseemann/barrnap (2013).

[46] Chan, P. P. & Lowe, T. M. tRNAscan-SE: searching for tRNA genes in genomic sequences. *Gene prediction**:**Methods in Mol. Biol*., **1962**, Humana, New York, NY, 161–177, 10.1007/978-1-4939-9173-0_1 (2019). 10.1007/978-1-4939-9173-0_1 PMC6768409 31020551

[47] Chan, P. P., Lin, B. Y., Mak, A. J. & Lowe, T. M. tRNAscan-SE 2.0: improved detection and functional classification of transfer RNA genes. *Nucleic Acids Res.* **49**(16), 9077–9096, 10.1093/nar/gkab688 (2021). 34417604 10.1093/nar/gkab688 PMC8450103

[48] *European Nucleotide Archive* https://identifiers.org/insdc.sra:ERP174591 (2025).

[49] *NCBI GenBank* https://identifiers.org/insdc.gca:GCA_965643865 (2025).

[50] Rupp, O., Kundu, S., Becker, A. & Das, M. Genome annotation files of Bambusa tulda. *Figshare*10.6084/m9.figshare.31037047 (2025).

[51] Emms, D. M. & Kelly, S. OrthoFinder: phylogenetic orthology inference for comparative genomics., **20**, 1–14. 10.1186/s13059-019-1832-y (2019). 10.1186/s13059-019-1832-y PMC6857279 31727128

[52] Simão, F. A., Waterhouse, R. M., Ioannidis, P., Kriventseva, E. V. & Zdobnov, E. M. BUSCO: assessing genome assembly and annotation completeness with single-copy orthologs. *Bioinformatics* **31**(19), 3210–3212, 10.1093/bioinformatics/btv351 (2015). 26059717 10.1093/bioinformatics/btv351

[53] Siri, J. N., Neufeld, E., Parkin, I. & Sharpe, A. Using Simulated Annealing to Declutter Genome Visualizations. *In FLAIRS* (pp. 201–204). Available online at https://github.com/jorgenunezsiri/accusyn (2020, May).

[54] Hao, Z. *et al*. RIdeogram: drawing SVG graphics to visualize and map genome-wide data on the idiograms. *PeerJ Comput. Sci.* **6**, e251, 10.7717/peerj-cs.251 (2020). 33816903 10.7717/peerj-cs.251 PMC7924719

